# Multiobjective reconfiguration of unbalanced distribution networks using improved transient search optimization algorithm considering power quality and reliability metrics

**DOI:** 10.1038/s41598-022-17881-x

**Published:** 2022-08-11

**Authors:** Mohana Alanazi, Abdulaziz Alanazi, Ahmad Almadhor, Zulfiqar Ali Memon

**Affiliations:** 1 Department of Electrical Engineering, College of Engineering, Jouf University, Sakaka, 72388 Saudi Arabia; 2 Department of Electrical Engineering, College of Engineering, Northern Border University, Ar’Ar, 73222 Saudi Arabia; 3 Department of Computer Engineering and Networks, College of Computer and Information Sciences, Jouf University, Sakaka, 72388 Saudi Arabia; 4 Department of Electrical and Computer Engineering, College of Engineering and Information Technology, Ajman University, Ajman, United Arab Emirates

**Keywords:** Engineering, Mathematics and computing

## Abstract

This paper proposes a new intelligent algorithm named improved transient search optimization algorithm (ITSOA) integrated with multiobjective optimization for determining the optimal configuration of an unbalanced distribution network. The conventional transient search optimization algorithm (TSOA) is improved with opposition learning and nonlinearly decreasing strategies for enhancing the convergence to find the global solution and obtain a desirable balance between local and global search. The multiobjective function includes different objectives such as power loss reduction, enhancement of voltage sag and unbalance, and network energy not supplied minimization. The decision variables of the reconfiguration problem including opened switches or identification of optimal network configuration are determined using ITSOA and satisfying operational and radiality constraints. The proposed methodology is implemented on unbalanced 13-bus and 118-bus networks. The results showed that the proposed ITSOA is capable to find the optimal network configuration for enhancing the different objectives in loading conditions. The results cleared the proposed methodology's good effectiveness, especially in power quality and reliability enhancement, without compromising the different objectives. Comparing ITSOA to conventional TSOA, particle swarm optimization (PSO), gray wolf optimization (GWO), bat algorithm (BA), manta ray foraging optimization (MRFO), and ant lion Optimizer (ALO), and previous approaches, it is concluded that ITSOA in improving the different objectives.

## Introduction

Recently, A distribution network is the ending stage of an electrical power supply system. It is where electrical energy is distributed to consumers. During power distribution, it can be lost in the form of a heat transfer current. The total power loss of large networks is very high^1,2^. Power losses directly affect the cost of operating a power system. Therefore, reducing losses and improving the distribution network characteristics is one of the most important tasks of distribution network operators. One of the least expensive ways to enhance the characteristics of the distribution network is reconfiguration^3,4^. Improving the network voltage conditions, reliability, and power quality are the most important objectives related to the operation problems. Few studies have considered these indices simultaneously in solving the reconfiguration problem^1–3^.

Moreover, in view of power quality, the study of voltage sag and unbalance, which is due to the increase in faults^5^ in the distribution network and unbalance load, has great importance. Load balancing or line flow balancing must be considered in the network reconfiguration because the unbalance load has adverse effects on distribution systems' performance. In the switching operation of optimal load balancing, unbalanced loads are transferred from high load feeders or transformers to low load feeders or transformers^1,3^. This increases the efficiency of network operation. On the other hand, achieving a better voltage profile changes the reconfiguration, network topology, and consequently, the values and load flow paths to prevent overload and losses due to long distribution lines. Moreover, another important objective of electricity distribution companies is to ensure the reliability of electricity delivered to customers as high as possible and provide electricity to customers entirely and without interruption^1^.

Therefore, the objective is to implement reconfiguration methods to improve the issues related to the reliability and power quality of the network. The network reconfiguration is a cost-effective method without the need for extra devices. Thus, the flow of load in the network can be modified, and consequently, the losses in the network can be reduced. Network reconfiguration has been known for a long time as a helpful method to improve the distribution network performance^2^. The distribution network is configured radially according to the operating advantages. Therefore, in the distribution network operation, the effect of reconfiguration on network indices such as losses, voltage, reliability, and power quality should be examined^1^. An optimal reconfiguration of the network can be determined by incorporating the desired objectives and considering operational and network radiality constraints. Since the number of switches in the network is large, the problem of network reconfiguration based on opened and closed network switches is complex. Solving it requires a robust optimization method^5–9^, with a high searching capability of the global solution. The use of classical and numerical methods is not logical due to the discrete nature of this problem and its constraints. In recent years it has been suggested to solve the reconfiguration problem using intelligent optimization methods^10,11^.

Various studies are conducted on the operation of distribution networks based on reconfiguration. So far, heuristic methods and conventional programming have been used for reconfiguration solving. Intelligent algorithms, such as iteration-based algorithms named artificial intelligence, have been widely welcomed to solve the reconfiguration problem^12,13^. In^14^, dynamic programming reduces network losses by considering load changes for solving the reconfiguration. It utilizes a genetic algorithm (GA) for reconfiguring the network to minimize loss^15^. In^16^, the network reconfiguration to reduce losses and voltage deviations is presented using a cuckoo search algorithm (CSA). The CSA ensures that network radiality is not affected during the reconfiguration process. The results confirmed the better performance of the CSA over the particle swarm optimization (PSO) method. In^17^, the reconfiguration of the balanced network is presented to minimize the losses and voltage deviations of the distribution network using the improved PSO. The results showed that network losses are significantly reduced by reconfiguration, and the voltage profile has also been improved. In^18^, balanced network reconfiguration is presented as a fuzzy multi-objective optimization with the objectives of the loss and voltage deviation minimization subject to the current constraint of the branches. In^19^, the balanced network reconfiguration is presented by minimizing the losses using the taboo search (TS) algorithm. In^20^, the reconfiguration problem is developed with a single purpose for reduction of the losses, and the ant colony search algorithm (CSA) is introduced. In^21^, a modified TS intelligent method is applied to solve the balanced network reconfiguration to minimize the active losses with the opening and closing of the network switches. In^22^, single objective bacterial foraging optimization (BFO) is applied to configure a balanced network considering loss minimization. The optimization results showed that the proposed single-objective algorithm significantly reduces network losses. In^23^, the optimization algorithm based on discrete teaching–learning based optimization (TLBO) has been applied to solve the reconfiguration problem integrated with distributed generations with minimizing losses and voltage profile enhancement. In^3^, improving the network power quality indices as unbalanced considering single-objective optimization is presented with ant lion optimizer (ALO). The results showed that the ALO could optimally determine the network configuration and improve the power quality. Using an exchange market and wild goats algorithm combination^24^, proposed a multi-objective optimization framework to optimize the configuration of a balanced network to reduce losses and improve reliability. In^25^, the network reconfiguration is studied by considering the types of distributed generations using improved PSO. The results are compared with GA, which showed the better capability of the improved PSO. In^26^, balanced network reconfiguration is investigated to reduce power/energy losses and minimize the network loading index using a combination of a heuristic method and GA. In^27^, balanced network reconfiguration is implemented to reduce the losses and enhance the network reliability criteria using modified GA. Using the bat algorithm (BA)^28^, investigates balanced network reconfiguration to improve reliability by minimizing the number of outages, the duration of outages, and the amount of unmet energy supply. In^29^, plant growth stimulation (PGS) is used to solve the combined problem of network reconfiguration and distributed generations for losses reduction and voltage profile enhancement. In^30^, the balanced network reconfiguration is presented to improve the power quality and reduce the voltage harmonics using the differential evolutionary (DE) method. In^31^, the unbalanced network reconfiguration is presented in terms of the effect on power quality and reduction of harmonics and unbalanced voltage distortion as single-objective optimization.

The research gap in the literature and the paper contributions are presented as follows:

- Based on the previous studies, it is evident that reconfiguration problems are more common in well-balanced distribution networks. In this paper an unbalanced distribution network is considered to configure the distribution network to achieve real-time and accurate operations.
- A literature review concluded that power quality, reliability, and power loss were not considered as part of a comprehensive reconfiguration study as a multi-objective optimization issue. Thus, in this paper multi-objective method based on these metrics are considered to solve the reconfiguration problem.
- In order to study the effect of reconfiguration more comprehensively, multiobjective functions should be considered to cover different aspects of the distribution network comprehensively. A weight coefficients method was used in some literature to reach an optimal solution. However, this method is not appropriate for determining the optimal global solution because the weight coefficients should be selected optimally. Nonetheless, in most studies, the results are based on trial and error. An idea has emerged as the multiobjective optimization^3,32,33^, whereby different objectives can be considered together as an objective function. Therefore, this study aims at bringing together the different yet essential objectives in the reconfiguration problem. Evolutionary algorithms based on the Pareto front have been one of the desirable methods for solving multi-criteria optimization problems. These methods have proven performance in solving problems with 2 or 3 objectives. There is difficulty in adding new objectives due to the loss of selection pressure of Pareto optimization, testing high-dimensional space density, anti-convergence, and exponentially increasing computation complexity. Therefore, Pareto-based evolutionary algorithms are used for a maximum of three objectives in the general objective function. Consequently, in this paper a multi-objective reconfiguration method is proposed considering four different objectives.
- Existing intelligent algorithms may be trapped in local optimal since the dimension and complexity of the problem increase, and they may converge rapidly. Hence, in this paper an improved optimization method based on the transient response of switched electrical circuits is used to overcome these shortcomings and achieve more precise network reconfiguration.

In this paper, a multiobjective optimization framework of the reconfiguration problem for the unbalanced distribution is performed to minimize the losses, enhance the voltage sag and unbalance (power quality enhancement) as well as reduce the network energy not supplied (reliability enhancement). A new intelligent algorithm named improved transient search optimization algorithm (ITSOA) is introduced to solve this challenging optimization problem. The conventional transient search optimization algorithm (TSOA)^34^ is modeled based on the transient response of switched electrical circuits. The performance of the conventional TSOA is improved with opposition learning and nonlinearly decreasing strategies to enhance the balance between local and global search, avoid getting trapped in local optimal, and increase the convergence efficiency. The optimization variable is considered as network opened switches (tie-lines) that are determined optimally using ITSOA. The proposed methodology is implemented on 13-bus typical network and also the IEEE 118-bus distribution network. The best or optimal unbalanced network configuration is identified in view of open and close switches based on the proposed method that leads to the lowest power losses, the best reliability, and power quality using ITSOA. The simulation results, including losses, voltage sag, voltage unbalance, and reliability indices, are compared without and with the network configuration using the ITSOA. The capability of the ITSOA is analyzed in comparison with the conventional TSOA and some high-ability methods such as particle swarm optimization (PSO)^35^ and grey wolf optimizer (GWO)^36^, manta ray foraging optimization (MRFO)^37^, bat algorithm (BA)^38^ and ant lion optimizer (ALO)^39^.

The contributions of the paper are listed as follows:

- A multi-objective optimization method to identify the optimal network configuration
- Considering different objectives based on losses, power quality and reliability for network reconfiguration
- Using of an improved transient search optimization algorithm (ITSOA) to configure the distribution network
- Superior performance of the ITSOA compared with PSO, GWO, MRFO, BA and ALO
- Lower convergence tolerance and higher convergence accuracy of the ITSOA in comparison with the other methods.

The paper is presented as follows; the objective function, constraint, and multiobjective function are described in “Problem formulation” section. In “Proposed ITSOA” section, the new, improved transient search optimization algorithm is described. In “Results and discussion” section, the results of the simulation and the findings are concluded in “Conclusion” section.

## Problem formulation

Multiobjective reconfiguration for unbalanced networks is formulated via ITSOA. So, the objective function and also operational constraints are presented in this section.

### Objective function

The objective function minimizes the losses, mitigates the voltage sag and voltage unbalance, and improves reliability by minimizing the ENS metric.

- Power loss
  Active power loss is calculated using the branches' currents and resistances^25,32^.
  1 $${P}_{loss}=\sum \limits_{k=1}^{n}{R}_{k}({I}_{k}{)}^{2}$$
  2 $${I}_{k}=\frac{{\lambda }_{i}-{\lambda }_{j}}{{R}_{k}+j{X}_{k}}$$
  where ${\lambda }_{i}$ and ${\lambda }_{j}$ refers to the voltages among buses *i* and *j, k* is the line among the buses *i* and *j*, ${R}_{k}$, ${X}_{k} \; \mathrm{and}$ ${I}_{k}$ are the resistance, reactance and *k*th line current, and *n* is the number of lines. The loss should be minimized in the proposed optimization problem.
- Voltage sag improvement
  The voltage sag is defined as decreased voltage in all network buses in the condition of voltage sag due to fault occurrence (0.5 cycles to 1 min). In this study, average voltage sag (${\lambda }_{sag.av}$) is calculated as the remaining voltage of the network buses during the voltage sag condition as follows^31^:
  3 $${\lambda }_{sag.av}=\frac{1}{m}\sum \limits_{j=1}^{m}\frac{1}{n}\sum \limits_{i=1}^{n}{\lambda }_{i}^{j}$$
  ${\lambda }_{i}^{j}$ refers to the voltage at bus *i* in fault conditions at bus *j*, *n* refers to the number of buses, and *m* indicates the possible faults number. The second objective function is considered as the minimization of voltage sag by minimizing $1/{\lambda }_{sag.av}$.
- Voltage unbalance improvement
  The voltage unbalance happens because the distribution network is unbalanced, which can be measured using the average voltage imbalance among the network buses^31^.
  4 $${\lambda }_{unb.av}=\frac{100}{n}\sum \limits_{i=1}^{n}\sum \limits_{i=a}^{c}\frac{\left|{\lambda }_{i}^{a}+{\alpha }_{2}{\lambda }_{i}^{b}+{\alpha }_{1}{\lambda }_{i}^{c}\right|}{\left|{\lambda }_{i}^{a}+{\alpha }_{1}{\lambda }_{i}^{b}+{\alpha }_{2}{\lambda }_{i}^{c}\right|}$$
  where, ${\alpha }_{1}=\mathrm{complex}(-\mathrm{0.5,0.866})$ and ${\alpha }_{2}=\mathrm{complex}(-0.5,-0.866)$. In this study, the voltage unbalance is considered to be minimized.
- Reliability Improvement
  The energy not supplied (ENS) is one of the critical indices of the reliability concept. In the event of a fault and the outage of the network lines, some network loads may be interrupted. The ENS is defined by^2^
  5 $$ENS=\sum \limits_{i=1}^{{N}_{br}}\sum \limits_{j=1}^{{N}_{l}}\left(O{R}_{i}\times {\psi }_{i}\times {\rm T}_{i}\times O{D}_{j}\right)$$
  where *N*_*br*_ indicates the number of network lines, *N*_*l*_ is interrupted loads number in a condition of the *i*th line outage, $O{R}_{i}$ is the failure rate per km in a year at line *i*, ${\psi }_{i}$ defines the length of line *i*, ${\rm T}_{i}$ is the time duration of fault *i* and $O{D}_{j}$ is the amount of unmet load under the fault *i*. So, to improve the reliability, the ENS should be minimized.

### Constraints

The multiobjective function is subjected to operational constraints to optimize the network configurations. The equality and inequality constraints are defined as follows^2,10,25,31^.

- Power balance equality
  6 $${P}_{i}+j{Q}_{i}={\lambda }_{ai}{I}_{ai}^{*}+{\lambda }_{bi}{I}_{bi}^{*}+{\lambda }_{ci}{I}_{ci}^{*}; \;\; \forall i=\mathrm{1,2},\ldots,n$$
- Voltage limits
  The voltage magnitude at each load bus should remain within allowable limits.
  7 $${\lambda }^{\mathrm{min}}\le {\lambda }_{pi}\le {\lambda }^{\mathrm{max}}; \;\;\forall p=a,b,c, \; \forall i=1, 2, \dots , n$$
- Thermal limit
  The current of the network lines should not be more than the tolerable current.
  8 $${I}_{pi}\le {I}_{pi}^{\mathrm{max}}; \; \forall p=a,b,c, \; \forall i=1, 2, \dots , n$$
- Unbalance voltage limit
  The bus's voltage unbalance must not be more than the allowable value (${\lambda }_{unb}^{\mathrm{max}}$).
  9 $$\frac{\left|{\lambda }_{Pos,i}\right|}{\left|{\lambda }_{Neg,i}\right|}\le {\lambda }_{unb}^{\mathrm{max}}; \; \forall i=1, 2, \dots , n$$
- Voltage sag limit
  The bus's voltage sag should not be less than the standard value (${\lambda }_{sag}^{\mathrm{min}}$).
  10 $${\lambda }_{sag,av}\ge {\lambda }_{sag}^{\mathrm{min}}$$
- Radiality constraint
  The network configuration should follow the radiality constraint in the reconfiguration solving (${n}^{\alpha }=n-1$). ${n}^{\alpha }$ refers to the closed lines number. So, inequality (11) should be satisfied to maintain network radiality in the reconfiguration process.
  11 $${\sum }_{j=1}^{L}\left|A(i,j)\right|\ge 1;\;\;\forall i=1, 2, \dots , n$$
  *A* refers to the bus incidence matrix in (11)^25^.

### Multiobjective optimization

This paper applies the fuzzy method to solve the optimization problem. The fuzzy decision is extracted by the intersection of fuzzy membership functions for different objectives. The Pareto front has been one of the desirable methods for solving multi-criteria optimization problems. This method has proven performance in solving problems with 2 or 3 objectives. Nevertheless, as reported in^33^, increasing the number of objectives is challenging. For example, the loss of selection pressure from Pareto dominance, determining high-dimensional space density and estimating the anti-convergence phenomenon. Therefore, Pareto-based evolutionary algorithms are used for a maximum of *three objectives* in the general objective function.

**Figure 1:**
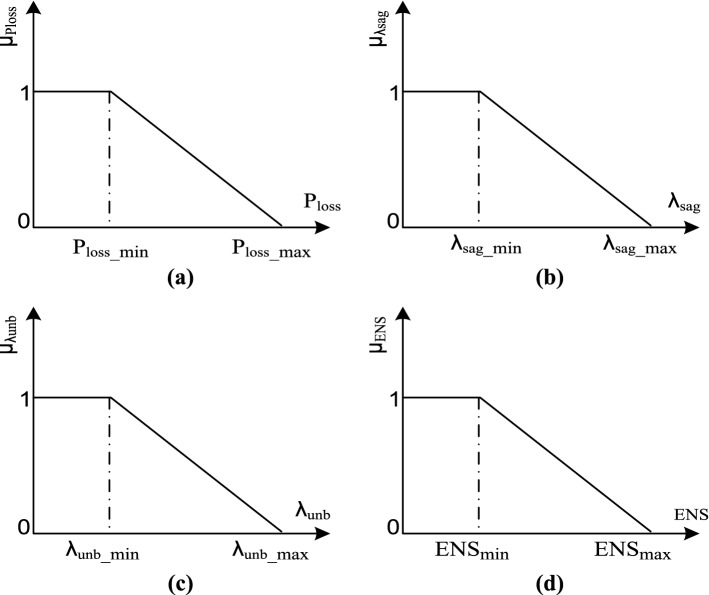
Fuzzy membership function applied for different objective functions (**a**) *P*_*loss*_, (**b**) ${\lambda }_{sag}$, (**c**) ${\lambda }_{unb}$, (**d**) ENS.

This paper develops a network reconfiguration model considering multiobjective optimization for *four critical objectives* in the general objective function. In this method, the first fuzzy membership function for each objective function (*μ*) should be defined to optimize the multiobjective problem. According to Fig. 1, the fuzzy membership functions for four objective functions are defined^2,32,33^. These fuzzy membership functions are formulated as follows:

12 $${\mu }_{Ploss}=\left\{\begin{array}{l}1;\quad { P}_{loss}\le {P}_{loss\_min}\\ \frac{{P}_{loss\_min}-{P}_{loss}}{{P}_{loss\_max}-{P}_{loss\_min}};\quad \;\;{P}_{loss\_min}\le {P}_{loss}\le {P}_{loss\_max}\\ 0;\quad { P}_{loss}\ge {P}_{loss\_max}\end{array}\right.$$

13 $${\mu }_{\lambda sag}=\left\{\begin{array}{l}1;\quad { \lambda }_{sag}\le {\lambda }_{sag\_min}\\ \frac{{\lambda }_{sag\_min}-{\lambda }_{sag}}{{\lambda }_{sag\_max}-{\lambda }_{sag\_min}};\quad \;\; {\lambda }_{sag\_min}\le {\lambda }_{sag}\le {\lambda }_{sag\_max}\\ 0;\quad { \lambda }_{sag}\ge {\lambda }_{sag\_min}\end{array}\right.$$

14 $${\mu }_{\lambda unb}=\left\{\begin{array}{l}1;\quad { \lambda }_{unb}\le {\lambda }_{unb\_min}\\ \frac{{\lambda }_{unb\_min}-{\lambda }_{unb}}{{\lambda }_{unb\_max}-{\lambda }_{unb\_min}};\quad \;\; {\lambda }_{unb\_min}\le {\lambda }_{sag}\le {\lambda }_{unb\_max}\\ 0;\quad { \lambda }_{unb}\ge {\lambda }_{unb\_min}\end{array}\right.$$

15 $${\mu }_{ENS}=\left\{\begin{array}{l}1;\quad { \lambda }_{ENS}\le {\lambda }_{ENS\_min}\\ \frac{{\lambda }_{ENS\_min}-{\lambda }_{ENS}}{{\lambda }_{ENS\_max}-{\lambda }_{ENS\_min}};\quad \;\; {\lambda }_{ENS\_min}\le {\lambda }_{ENS}\le {\lambda }_{ENS\_max}\\ 0;\quad { \lambda }_{ENS}\ge {\lambda }_{ENS\_min}\end{array}\right.$$

The minimum value of each objective is obtained using the network reconfiguration solving using the ITSOA. The maximum objective function value is found in the analysis of the base network. The nearest maximum value to the minimum value clears the higher importance degree of the objective. So, the nearest objective value to the maximum value is lower *µ*. At least one fuzzy index must be maximized to optimize the objective functions. In other words, the objective functions are minimized simultaneously, and all the objective functions are close to their minimum value. This method is formulated as follows^2,32,33^

16 $$\mu (x)=\mathrm{min}\left({\mu }_{{P}_{loss}}(x),{\mu }_{{\lambda }_{sag}}(x),{\mu }_{{\lambda }_{unb}}(x),{\mu }_{ENS}(x)\right)$$

The membership values define the satisfaction degree for any objective function. The high objective function has a low membership value and vice versa. So, the multiobjective problem is modified to a maximization problem as follows:

17 $$\mathrm{max }\left\{\mu (x)\right\}$$

The paper presents fuzzy membership functions for four objectives, as shown in Fig. 1.

## Proposed ITSOA

The proposed optimization method to find the optimal configuration of the unbalanced networks is presented in the following.

### Overview of transient search optimization algorithm (TSOA)

The TSOA algorithm is an innovative approach inspired by electrical phenomena, which integrates two energy-saving components. It is based on the transient response (TR) of switched electrical circuits (SECs). These elements are resistor, inductor and capacitor. The TSOA exploration capability inspires the response of the R-L-C circuit in under-damped transient conditions. So, the TSOA exploitation inspires the exponential decadence of the R-C circuit response in transient conditions. Hence, the response based voltage related to the capacitors in circuits of R-C and R-L-C are defined as follows^34^:

18 $${v}_{1}(t)={v}_{1}(\infty )+({v}_{1}(0)-{v}_{1}(\infty )){e}^{\frac{-t}{{R}_{1}{C}_{1}}}$$

19 $${v}_{2}(t)={e}^{\frac{-{R}_{2}t}{2L}}({\beta }_{1}\mathrm{cos}(2\pi {f}_{d}t)+{\beta }_{2}\mathrm{sin}(2\pi {f}_{d}t))+{v}_{2}(\infty );\quad \;\;\mathrm{if} (\frac{{R}_{2}}{2L}{)}^{2}<\frac{1}{L{C}_{2}}$$

R, L and C refer to resistance, inductor and capacitors, respectively. ${v}_{1}(t)$ and ${v}_{2}(t)$ are the response of R-C and R-L-C circuits, respectively. Also, *fd* refers to the damping frequency, and *B1* and *B2* indicate constant numbers.

**Figure 2:**
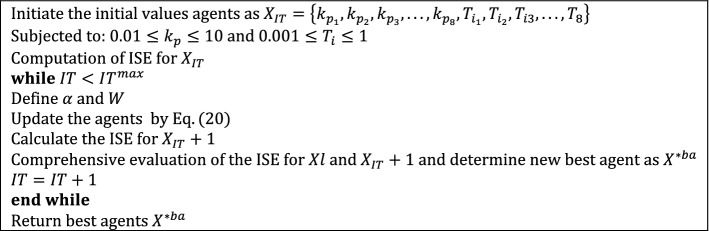
Pseudo-code of the TSOA.

So, the voltage response of the mentioned circuit in Eqs. (18) and (19) are applied for modeling the TSOA. In the TSOA model (Eq. (20)), the R, L and C (*R*1, *R*2, *C*1, *C*2, and *L*) in ${v}_{1}(t)$ and ${v}_{2}(t)$ equations are converted to random numbers as *U* and $\alpha$. This random characteristic is desirable for optimization methods. The decision variables in TSOA are considered as search agents *X*_*IT*_ + *1* and *X*_*IT*_, which are equivalent to *v*(*t*) and *v*(*0*) variables in ${v}_{1}(t)$ and ${v}_{2}(t)$ equations. Also, the X^*ba^ is a variable that is introduced as the best agent and equivalent to *v*(∞). Moreover, in the equation of ${v}_{2}(t)$, ${\beta }_{1}={\beta }_{2}=$|*X*_*IT*_ − *W. X*_IT_^*ba^| is established where *U* random number is defined by *U* = *k* × *rm*_2_ × *a* + 1 that *k* refers to an actual number and *a* = 2 − 2 × *IT*/*IT*^*max*^ that *IT*^*max*^ indicates maximum iterations number and *rm*_2_ refers to a number randomly between zero and one. The *r*_1_ is applied for balancing the exploration and exploitation phases with *rm*_*1*_ ≥ 0.5 and *rm*_*1*_ < 0.5, respectively, in the TSOA^34^.

20 $${X}_{IT+1}=\left\{\begin{array}{ll}{X}_{IT}^{*ba}+\left({X}_{IT}-U\times {X}_{IT}^{*ba}\right){e}^{-\alpha }; & \quad r{m}_{1}<0.5\\ {X}_{IT}^{*ba}+{e}^{-\alpha }[\mathrm{cos}(2\pi \alpha )+\mathrm{sin}(2\pi \alpha )]\left|{X}_{IT}-U\times {X}_{IT}^{*ba}\right|; & \quad r{m}_{1}\ge 0.5\end{array}\right.$$

where $\alpha$ = 2 × *a* × *rm*_3_ − *a*, and *rm*_3_ refer to real numbers randomly among zero and one., the pseudo-code of the TSOA is shown in Fig. 2.

### Overview of improved TSOA (ITSOA)

In this study, the opposition learning strategy (OLS)^40^ is applied to improve the convergence efficiency and balance the exploration and exploitation of the TSOA. These algorithms may get stuck in local optimality due to the selection of the optimization variable randomly and lack of desirable knowledge of domain search and essential criteria. Also, these methods are time-consuming due to the difference between the initial and optimal solutions. Even if these algorithms reach the global optimal, they may not have a good convergence speed and accuracy. TSOA uses OLS to find an alternative to the current solution. This approach allows the optimal solution to be approached and reached more quickly. Consequently, the convergence speed increases. Suppose *X* is in $\left[\eta ,\lambda \right]$. Then the opposite point is $\overline{X}=\eta +\lambda -X.$ $X=\left({X}_{1},{X}_{2},\ldots,{X}_{d}\right)$ is a point in search space with d dimension, where ${X}_{1},{X}_{2},\ldots,{X}_{d}\in R$ and ${X}_{i}\in \left[{\eta }_{i},{\lambda }_{i}\right]; \forall i\in \left\{\mathrm{1,2},\ldots,d\right\}.$ The opposite point based on its components is as follows:

21 $$\overline{X}={\eta }_{i}+{\lambda }_{i}-{X}_{i},\overline{X}=\left({\overline{X}}_{1},{\overline{X}}_{2},\ldots,{\overline{X}}_{d}\right)$$

So, the initial population is generated using the OLS.

Also, a nonlinearly decreasing strategy (NDS)^41^ is applied to improve the TSOA performance in global and local exploration and achieve a desirable balance between global convergence and convergent efficiency. Search performance can be improved with the changes in the coefficient of ${\partial }^{NDS}$. The ${\partial }^{NDS}$ reduced nonlinearly from ${\partial }_{\mathrm{max}}^{NDS}$ to ${\partial }_{\mathrm{min}}^{NDS}$. The more significant value of ${\partial }^{NDS}$ helps better global search capability. Besides, the smaller ${\partial }^{NDS}$ enhances global search capability in the exploration phase. This strategy is modeled by

22 $${\partial }^{NSD}={\partial }_{\mathrm{max}}^{NDS}-\frac{IT\times \left({\partial }_{\mathrm{max}}^{NDS}-{\partial }_{\mathrm{min}}^{NDS}\right)}{{IT}^{\mathrm{max}}}\times \mathrm{sin}\left(\frac{IT\times \pi }{2\times {IT}^{\mathrm{max}}}\right)$$

where, ${\partial }_{\mathrm{max}}^{NDS}$ and ${\partial }_{\mathrm{min}}^{NDS}$ refer to the upper and lower limit of coefficient ${\partial }^{NDS}$, respectively.

So (22) is modified by

23 $${X}_{IT+1}=\left\{\begin{array}{ll}{\partial }^{NSD}{X}_{IT}^{*ba}+\left({X}_{IT}-U{X}_{IT}^{*ba}\right){e}^{-\alpha }; & \quad {rm}_{1}<0.5\\ {\partial }^{NSD}{X}_{IT}^{*ba}+\left|{X}_{IT}-U{X}_{IT}^{*ba}\right|{e}^{-\alpha }\left(\mathrm{cos}\left(2\pi \alpha \right)+\mathrm{sin}(2\pi \alpha )\right); & \quad {rm}_{1}\ge 0.5\end{array}\right.$$

**Figure 3:**
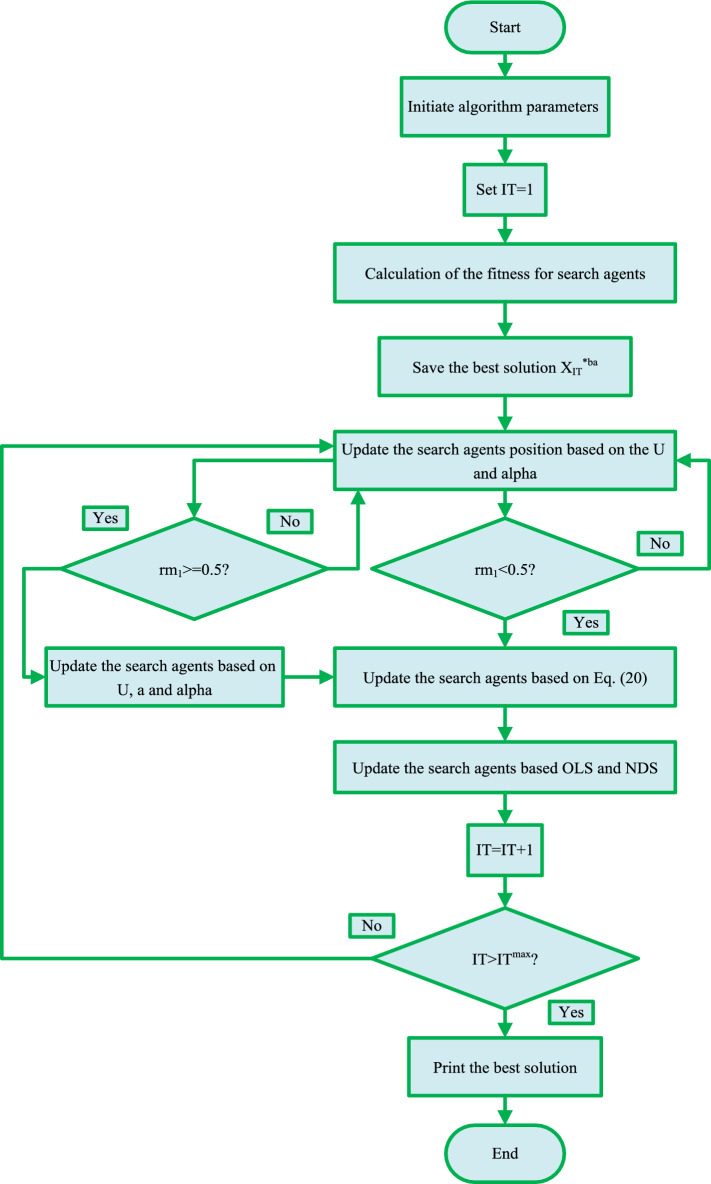
Flowchart of ITSOA.

The ITSOA can have high global search capability in pre-search, and finally, its convergence rate is fast. The flowchart of the proposed ITSOA is shown in Fig. 3.

### Comprehensive analysis of ITSOA

In this section, the capability of the ITSOA is investigated for some benchmark test functions that are available^42^. The benchmark test functions are presented in Table 1, as uni-modal (F1–F7), multi-modal (F8–F13) and fixed-dimension multimodal (F14–F23) in the prior studies. The uni-modal functions have only one global optimal, and the multi-modal functions have many local optimal. The ITSOA superiority is evaluated compared to the conventional TSOA and the results of Ref.^42^ obtained by the m-sine cosine algorithm (m-SCA). The ITSOA is achieved to the best value in all test functions with more substantial competitiveness compared with the TSOA and m-SCA. The mean and STD values of test functions obtained using different algorithms are presented in Table 2. The obtained results showed that the ITSOA achieves better values of the test functions and is very competitive compared to the other algorithm. These figures proved the competitive performance of the ITSOA compared with the conventional TSOA.

**Table 1:** The benchmark problems.

Test function	Dimensions	Range	F_min_
${F}{1}\left(x\right)={\sum }_{i=1}^{D}{x}_{i}^{2}$	30	[− 100, 100]	0
${F}{2}\left(x\right)={\sum }_{i=1}^{D}\left|{x}_{i}\right|+{\prod }_{i=1}^{D}\left|{x}_{i}\right|$	30	[− 10, 10]	0
${F}{3}\left(x\right)={\sum }_{i=1}^{D}{\left({\sum }_{j=1}^{i}{x}_{j}\right)}^{2}$	30	[− 100, 100]	0
${F}{4}\left(x\right)={\mathit{max}}_{i}\left\{\left|{x}_{i}\right|,1\le i\le D\right\}$	30	[− 100, 100]	0
${F}{5}\left(x\right)={\sum }_{i=1}^{D-1}\left[100({x}_{i}^{2}-{x}_{i+1}{)}^{2}+({x}_{i}-1{)}^{2}\right]$	30	[− 30, 30]	0
${F}{6}\left(x\right)={\sum }_{i=1}^{D}{\left(\left[{x}_{i}+0.5\right]\right)}^{2}$	30	[− 100, 100]	0
${F}{7}\left(x\right)={\sum }_{i=1}^{D}i{x}_{i}^{4}+random(\left.0, 1\right)$	30	[− 1.28, 1.28]	0
${F}{8}\left(x\right)={\sum }_{i=1}^{D}-{x}_{i}\mathit{sin}\left(\sqrt{\left|{x}_{i}\right|}\right)$	30	[− 500, 500]	− 418.9829 × *D*
${F}{9}\left(x\right)={\sum }_{i=1}^{n}\left[{x}_{i}^{2}-10\mathit{cos}\left(2\pi {x}_{i}\right)+10\right]$	30	[− 5.12, 5.12]	0
${F}{10}\left(x\right)=-20\mathit{exp}(-0.2\sqrt{{D}^{-1}{\sum }_{i=1}^{D}{x}_{i}^{2}}) -{\exp}\left({D}^{-1}{\sum }_{i-1}^{D}\mathit{cos}\left(2\pi {x}_{i}\right)\right)+20+e$	30	[− 32, 32]	0
${F}{11}\left(x\right)=\frac{1}{4000}{\sum }_{i=1}^{D}{x}_{i}^{2}-{\prod }_{i=1}^{D}\mathit{cos}\frac{{x}_{i}}{\sqrt{i}}+1$	30	[− 600, 600]	0
$${F}{12}\left(x\right)=\frac{\pi }{30}\left\{\begin{array}{l}10{\mathit{sin}}^{2}\left(\pi {y}_{1}\right)\\ +{\sum }_{i=1}^{D-1}{\left({y}_{i}-1\right)}^{2}\left[1+10{\mathit{sin}}^{2}\left(\pi {y}_{i}+1\right)\right]\\ +{\left({y}_{D}-1\right)}^{2}\end{array}\right\}+{\sum }_{i=1}^{D}u\left({x}_{i},\alpha ,k,m\right)$$ $$u\left({x}_{i},\alpha ,k,m\right)=\left\{\begin{array}{l}k{\left({x}_{i}-\alpha \right)}^{m},{x}_{i}>\alpha ,\\ 0,-\alpha \le {x}_{i}\le \alpha ,\\ k{\left(-{x}_{i}-\alpha \right)}^{m},{x}_{i}<-\alpha .\end{array}\right.$$ where ${y}_{i}=1+\left({x}_{i}+1\right)/4$*, ɑ* = 5, *k* = 100 and *m* = 4	30	[− 50, 50]	0
$${F}{13}\left(x\right)=0.1\left\{\begin{array}{l}{\mathit{sin}}^{2}\left(3\pi {x}_{1}\right)\\ +{\sum }_{i=1}^{D}{\left({x}_{i}-1\right)}^{2}\left[1+{\mathit{sin}}^{2}\left(3\pi {x}_{i}+1\right)\right]\\ +{\left({x}_{D}-1\right)}^{2}\left[1+{\mathit{sin}}^{2}\left(2\pi {x}_{D}\right)\right]\end{array}\right\}+{\sum }_{i=1}^{D}u\left({x}_{i},\alpha ,k,m\right).$$	30	[− 50, 50]	0

$F{14} \left( x \right) = \left[ {\frac{1}{500} + \mathop \sum \limits_{j = 1}^{25} \frac{1}{{j + \mathop \sum \nolimits_{i = 1}^{2} (x_{i} - a_{ij} )^{6} }}} \right]^{ - 1}$	2	[− 65, 65]	0.998
${F}{15}\left(x\right)={\sum }_{i=1}^{11}{\left[{a}_{i}-\frac{{x}_{1}\left({b}_{i}^{2}+{b}_{i}{x}_{2}\right)}{{b}_{i}^{2}+{b}_{i}{x}_{3}+{x}_{4}}\right]}^{2}$	4	[− 5, 5]	0.0003
${F}{16}\left(x\right)=4{x}_{1}^{2}-2.1{x}_{1}^{4}+\frac{1}{3}{x}_{1}^{9}+{x}_{1}{x}_{2}-4{x}_{2}^{2}+4{x}_{2}^{4}$	2	[− 5, 5]	− 1.0316
${F}{17}\left(x\right)={\left({x}_{2}-\frac{5.1}{4{\pi }^{2}}{x}_{1}^{2}+\frac{5}{\pi }{x}_{1}-6\right)}^{2}+10\left(1-\frac{1}{8\pi }\right){\mathit{cos}x}_{1}+10$	2	[− 5, 5]	0.398
$$\begin{aligned}{F}{18}\left(x\right)& =\left[1+{\left({x}_{1}+{x}_{2}+1\right)}^{2}\left(19-14{x}_{1}+3{x}_{1}^{2}-14{x}_{2}+6{x}_{1}{x}_{2}+3{x}_{2}^{2}\right)\right] \\ & \quad \times \left[30+{\left(2{x}_{1}-3{x}_{2}\right)}^{2}\times \left(18-32{x}_{1}+12{x}_{1}^{2}+48{x}_{2}-36{x}_{1}{x}_{2}+27{x}_{2}^{2}\right)\right]\end{aligned}$$	2	[− 2, 2]	3
${F}{19}\left(x\right)=-{\sum }_{i=1}^{4}{c}_{i}\mathit{exp}\left(-{\sum }_{j=1}^{3}{a}_{ij}{\left({x}_{j}-{p}_{ij}\right)}^{2}\right)$	3	[1, 3]	− 3.86
${F}{20}\left(x\right)=-{\sum }_{i=1}^{4}{c}_{i}\mathit{exp}\left(-{\sum }_{j=1}^{6}{a}_{ij}{\left({x}_{j}-{p}_{ij}\right)}^{2}\right)$	6	[1, 3]	− 3.32
${F}{21}\left(x\right)=-{\sum }_{i=1}^{5}\left.(x-{a}_{i}\right)(x-{a}_{i}{)}^{T}+{c}_{i}{]}^{-1}$	4	[0, 10]	− 10.1532
${F}{22}\left(x\right)=-{\sum }_{i=1}^{7}\left.(x-{a}_{i}\right)(x-{a}_{i}{)}^{T}+{c}_{i}{]}^{-1}$	4	[0, 10]	− 10.4028
${F}{23}\left(x\right)=-{\sum }_{i=1}^{10}\left.(x-{a}_{i}\right)(x-{a}_{i}{)}^{T}+{c}_{i}{]}^{-1}$	4	[0, 10]	− 10.5363

**Table 2:** The Mean and Std. values of test functions using ITSOA, and m-SCA^42^ with *D* = 30.

F	ITSOA		m-SCA	
	Ave	Std	Ave	Std
F1	4.12E−5	4.87E−4	5.70E−03	2.63E−02
F2	7.48E−6	6.06E−4	9.11E−04	1.90E−03
F3	8.17E + 2	4.15E+2	8.48E+02	5.49E+02
F4	6.98E−1	4.26E−1	7.07E−01	5.37E−01
F5	25.97E+00	8.51E+00	29.5658	2.43E+00
F6	1.06E+00	5.44E−1	1.24E+00	5.12E−01
F7	1.53E−02	6.82E−03	1.95E−02	6.87E−03
F8	− 4.18E+03	2.35E+02	− 4.26E+03	2.88E+02
F9	6.38E+01	4.90E+01	7.81E+01	5.21E+01
F10	4.309E−5	7.16E−04	3.36E−03	8.06E−03
F11	3.25E−2	5.92E−03	3.84E−02	7.15E−02
F12	1.34E−01	6.77E−02	1.45E−01	8.19E−02
F13	1.27E+00	3.13E−01	1.41E+00	3.94E−01
F14	1.01 E+00	0.00E+00	1.03E+00	1.81E−01
F15	5.03E−04	0.89E−04	5.10E−04	1.00E−04
F16	− 1.03E+00	0.00E+00	− 1.03E+00	0.00E+00
F17	3.98E−01	0.00E+00	3.98E−01	0.00E+00
F18	3.00E+00	6.76E−16	3.00E+00	1.00E−04
F19	− 3.86E+00	2.28E−15	− 3.86E+00	2.00E−04
F20	− 3.31E+00	0.48E−02	− 3.31E+00	0.51E−02
F21	− 9.98E+00	1.74E−01	− 9.94E+00	1.99E−01
F22	− 1.02E+01	2.05E−01	− 1.01E+01	2.10E−01
F23	− 1.03E+01	0.96E−01	− 1.03E+01	1.13E−01

### Implementation of ITSOA

Friedman test^43,44^ is performed to better show the performance of the ITSOA on the average value compared with m-SCA and the results are given in Table 3. Based on the results obtained, the ITSOA has effectively defeated the m-SCA, which indicates the strength of this algorithm.

**Table 3:** Friedman ranks of ITSOA and m-SCA for all test functions.

Function	m-SCA	ITSOA
F1	2	1
F2	2	1
F3	2	1
F4	2	1
F5	2	1
F6	2	1
F7	2	1
F8	2	1
F9	2	1
F10	2	1
F11	2	1
F12	2	1
F13	2	1
F14	2	1
F15	2	1
F16	2	1
F17	2	1
F18	2	1
F19	2	1
F20	2	1
F21	2	1
F22	2	1
F23	2	1
AVG-Rank	2.00	1.00
Final Rank	2	1

The Wilcoxon test is also used to present the performance of the ITSOA compared to the m-SCA. Based on the results obtained in Table 4, the CVSO is decisively superior to the m-SCA and this proposed algorithm is more competitive.

**Table 4:** The competitive results of Wilcoxon’s test.

Corresponding algorithm	ITSOA versus
	*p*-values
m-SCA	9.7505e−9

The ITSOA is developed for solving the multiobjective optimal network reconfiguration. The ITSOA implementation is presented below to determine the optimum network configuration.

Step 1: The distribution network is radial in normal conditions. However, the network has tie-lines that are open in standard conditions and can insert into the network by maintaining the network radiality and observing the other constraints of the problem. Another line should be opened in order to maintain the network's radiality.
Step 2: Typically, the lines (which should not be identical) are chosen at random. The opened lines (which should not be the same) are selected randomly. In this condition, the network radiality and operation constraints should be satisfied.
Step 3: The objective functions are calculated for opened lines satisfying the operational constraints.
Step 4: The ITSOA populations are updated.
Step 5: The objective function is calculated for new ITSOA populations. If the objective function value is lower than the ones obtained in Step 3, it should be replaced with the old value.
Step 6: If convergence criteria (running the maximum iterations of the ITSOA) are satisfied, go to Step 7; otherwise, go to step 4.
Step 7: The ITSOA is stopped.

## Results and discussion

The unbalanced 13-bus Kodaband-Loo network and IEEE 118-bus distribution networks are used to validate the proposed methodology. The Kodaband-Loo distribution network has an active load of 10.536 kW and 5.992 kvar, 63/20 kV. The unbalanced 13-bus Khodabandeh-loo network has five lines between the buses 13–4, 6–9, 13–12, 12–10 and 9–10, lines 13 to 17 of the network^45^. The 118-bus network also has 118 buses and 117 lines. The 118-bus network has 15 tie lines that these lines are Lines 118 to 132, respectively between buses 46–27, 17–27, 8–24, 43–54, 62–49, 62–37, 9–40, 58–96, 91–73, 88–75, 77–99, 83–108, 86–105, 110–118, 25–35^45^. The data of the 118-bus network is derived from^39^. The configuration results via the ITSOA are given subjected to loading variations. The results of the ITSOA in the reconfiguration problem are compared with conventional TSOA and some well-known and powerful algorithms such as PSO, GWO, MRFO, ALO and BA. The algorithm population is considered 500, and also the iteration number is selected 300 based on the user experience and different running the simulations.

### 13-bus typical unbalanced network

**Figure 4:**
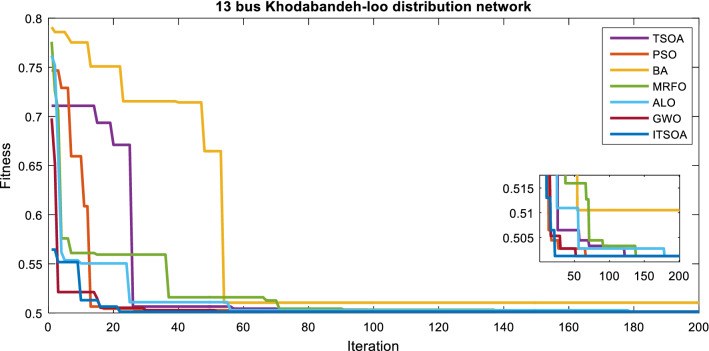
Convergence process of the algorithms in multiobjective reconfiguration solution, unbalanced 13-bus network.

Simulation results are presented for a multi-objective reconfiguration to minimize loss, improve power quality, and increase the reliability of a typical feeder. The convergence process of the ITSOA in comparison with different algorithms is presented in Fig. 3. According to Fig. 4, the high convergence speed of ITSOA is proved in lower iteration and convergence tolerance also with the lowest objective function. In Table 5, the numerical results are demonstrated for the 13-bus network.

**Table 5:** Simulation results of multiobjective reconfiguration unbalanced 13-bus network.

Method	Solution	Loss (kW)	$\lambda$_sag_ (p.u)	$\lambda$_un_ (%)	ENS (MWh/yr)
Initial	13, 14, 15, 16, 17	175.58	0.980	0.810	4.346
ITSOA	6, 7, 9, 12, 13	16.687	0.383	0.406	2.603
TSOA	6, 7, 9, 13, 15	16.905	0.394	0.4081	2.737
PSO	6, 7, 9, 13, 15	16.905	0.394	0.4060	2.737
BA	5, 7, 12, 13, 17	17.595	0.409	0.4133	2.817
GWO	6, 7, 9, 13, 15	16.905	0.394	0.4060	2.737
MRFO	6, 8, 9, 10, 13	17.051	0.399	0.4117	2.762
ALO	6, 7, 9, 13, 15	16.905	0.394	0.4060	2.737

The obtained results showed that, according to Table 6, after optimal reconfiguration, the objectives of loss, power quality indices and reliability of the 13-bus network are enhanced. The value of network losses in the base case in nominal load is 175.58 kW, which after reconfiguration reached 116.687 kW. Switches 6, 7, 9, 12, 13 are determined as opened switches by ITSOA. Also, the power quality indices and ENS values without network optimization are 0.980 p.u, 0.81% and 4.348 kWh, respectively, which decreased to 0.383 p.u, 0.4060%, and 2.603 kWh after multiobjective reconfiguration, respectively.

**Table 6:** Statistic Analysis of methods for multiobjective problem of 13-bus unbalanced network.

Algorithms	Best	Mean	Worst	STD
ITSOA	0.50124	0.50139	0.50257	0.00045953
TSOA	0.50270	0.50198	0.50329	0.00083409
PSO	0.50270	0.50233	0.50464	0.0011035
BA	0.51652	0.53445	0.57564	0.026266
GWO	0.50270	0.50154	0.50277	0.00062865
MRFO	0.50630	0.51992	0.73949	0.060781
ALO	0.50270	0.50133	0.50270	0.0006085

**Figure 5:**
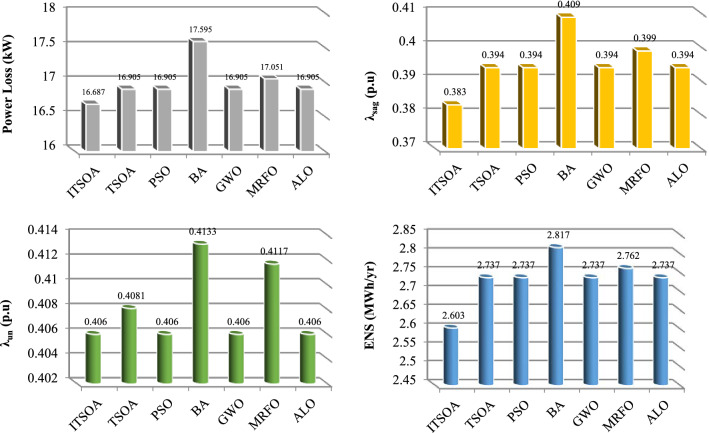
The optimal objective values obtained with different algorithms for the unbalanced 13-bus network.

**Figure 6:**
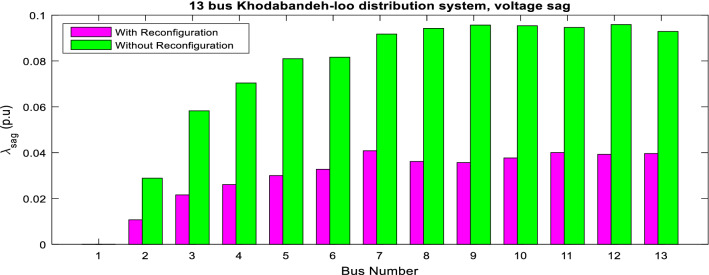
Voltage sag of 13-bus network via ITSOA with and without reconfiguration.

**Figure 7:**
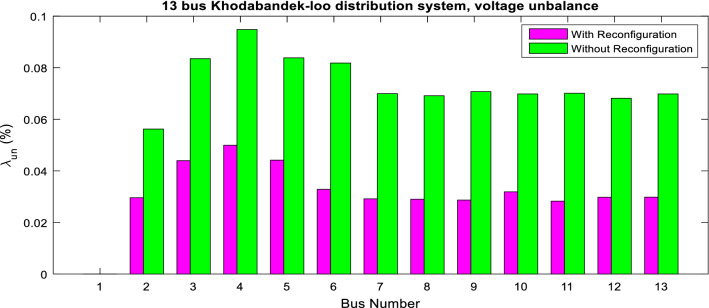
Voltage unbalance of 13-bus network via ITSOA with and without reconfiguration.

The superiority of the ITSOA in problem solution with achieving the lowest value of losses, power quality indices and ENS is illustrated compared with the other algorithms for the 13-bus network in Fig. 5. Also, the statistic test of different methods' capability for multiobjective configuration of the unbalanced 13-bus network is given in Table 6. The results showed better optimization performance of ITSOA compared with the TSOA, PSO, BA, GWO, MRFO and ALO methods in obtaining a better objective function value with less STD. Moreover, the power quality indices of the 13-bus network are illustrated in Figs. 6 and 7, respectively. According to Figs. 6 and 7, power quality is improved by determining optimal network configuration based on multiobjective functions.

**Figure 8:**
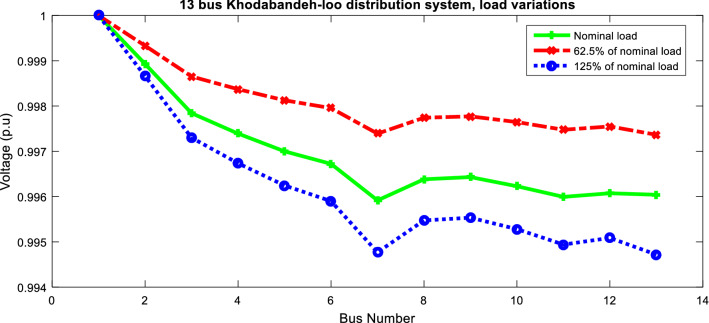
Voltage profile of unbalanced 13-bus network in different loading using ITSOA after reconfiguration.

The effect of load variation of the unbalanced 13-bus network is studied in the reconfiguration results. In Table 7, the results of changing the demand are presented. The results showed that increasing the network load causes increase in power loss, voltage sag, voltage unbalance and energy not supplied values. So it can be said that the reliability and power quality are also affected by network demand. The voltage profile of the 13-bus network is shown in Fig. 8 in different loading conditions. It can be seen that the network voltage deviation is reduced with load decreasing and vice versa.

**Table 7:** Effect of changing the demand for the multiobjective problem of the unbalanced 13-bus network via ITSOA.

Method	Solution	Loss (kW)	$\lambda$_sag_ (p.u)	$\lambda$_un_ (%)	ENS (MWh/yr)
62.5% of the nominal load	6, 7, 9, 13, 15	65.858	0.246	0.2530	1.710
Initial	13, 14, 15, 16, 17	125.45	0.690	0.601	2.954
Nominal load	6, 7, 9, 12, 13	116.687	0.383	0.4060	2.603
Initial	13, 14, 15, 16, 17	175.58	0.980	0.810	4.346
125% of nominal load	6, 7, 9, 13, 15	152.117	0.493	0.5085	3.421
Initial	13, 14, 15, 16, 17	212.06	1.164	0.987	5.046

### 118-bus unbalanced network

**Figure 9:**
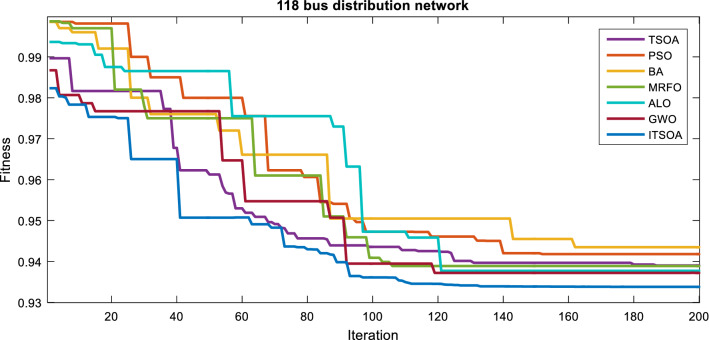
Convergence process of the algorithms in multiobjective reconfiguration solution, 118-bus network.

The simulation results for an unbalanced 118-bus distribution network are demonstrated. The base losses, voltage sag, ENS, and voltage unbalance are obtained at 1322.470 kW, 2.549 p.u, 128.274 kWh and 23.098%. The convergence process of TSOA, PSO, GWO, MRFO, ALO, BA, and ITSOA is shown in Fig. 9. The results showed that the ITSOA is achieved to the best objective function value and converged with less tolerance than the other algorithms. The results showed that the better performance of the proposed method is in the reconfiguration of large networks. The optimization procedure in a large distribution network is complex, which can be a good test for evaluating the performance of the optimization methods.

**Figure 10:**
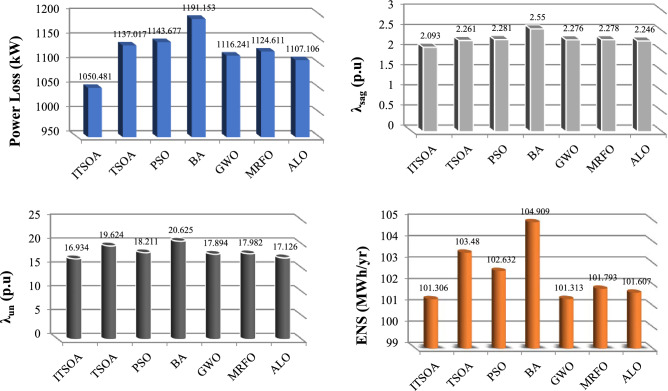
The optimal optimal objective values obtained with different algorithms for the unbalanced 118-bus network.

In Table 8, the simulation results of multiobjective reconfiguration based on ITSOA for the nominal unbalanced 118-bus system demand are depicted. The switches of 118, 119, 120, 121, 122, 123, 124, 125, 126, 127, 128, 129, 130, 131, 132 are open in base condition. Best configuration of 118-bus network is with opened switched as 14, 23, 33, 39, 43, 47, 71, 85, 87, 95, 107, 108, 121, 123, 128 determined optimally by ITSOA. The losses, power quality indices and ENS are 1050.481 kW, 2.0930 p.u, 16.934%, and 101.306 kWh, respectively. The superiority of the ITSOA in problem solutions with achieving better indices in comparison without reconfiguration is illustrated compared with the other algorithms for the 118-bus network in Fig. 10. Table 9 also gives a statistic test of methods capability for multiobjective reconfiguration of the 118-bus system. The results proved that the Best value of ITSOA is better than the other algorithm and also achieved less STD in comparison with the other algorithms.

**Table 8:** Simulation results of multiobjective reconfiguration 118-bus unbalanced network.

Method	Solution	Loss (kW)	$\lambda$_sag_ (p.u)	$\lambda$_un_ (%)	ENS (MWh/yr)
Initial	118, 119, 120, 121, 122, 123, 124, 125, 126, 127, 128, 129, 130, 131, 132	1322.47	2.549	23.098	128.274
ITSOA	14, 23, 33, 39, 43, 47, 71, 85, 87, 95, 107, 108, 121, 123, 128	1050.481	2.0930	16.934	101.306
TSOA	21, 25, 33, 39, 45, 48, 60, 68, 76, 109, 121, 125, 126, 129, 130	1137.017	2.261	19.624	103.480
PSO	15, 20, 33, 40, 43, 47, 70, 76, 85, 86, 95, 105, 109, 121, 123	1143.677	2.281	18.211	102.632
BA	8, 23, 26, 33, 41, 46, 48, 73, 76, 89, 102, 105, 123, 126, 129	1191.153	2.550	20.625	104.909
GWO	23, 26, 34, 39, 42, 53, 60, 71, 73, 95, 96, 109, 122, 129, 130	1116.241	2.276	17.894	101.313
MRFO	23, 27, 33, 41, 53,62, 71, 76, 123, 125, 126, 129, 130, 131, 132	1124.611	2.278	17.982	101.793
ALO	23, 24, 26, 39, 46, 48, 53, 57, 60, 67, 87, 96, 109, 129, 130	1107.106	2.246	17.126	101.607

**Table 9:** Statistic test of methods capability for multiobjective problem of unbalanced 118-bus network.

Method	Best	Mean	Worst	STD
ITSOA	0.9338	0.94501	0.95371	0.0029254
TSOA	0.9390	0.9485	0.95474	0.0035381
PSO	0.9418	0.9488	0.95891	0.003880
BA	0.9435	0.9512	0.96214	0.005566
GWO	0.9372	0.9483	0.95684	0.003159
MRFO	0.9389	0.9486	0.95706	0.003344
ALO	0.9365	0.9478	0.95592	0.003137

**Figure 11:**
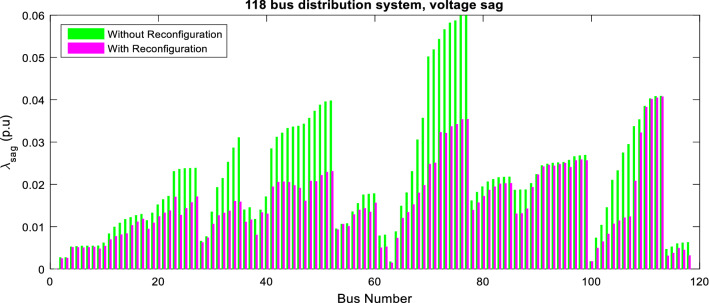
Voltage sag of 118-bus network via ITSOA with and without optimal configuration.

**Figure 12:**
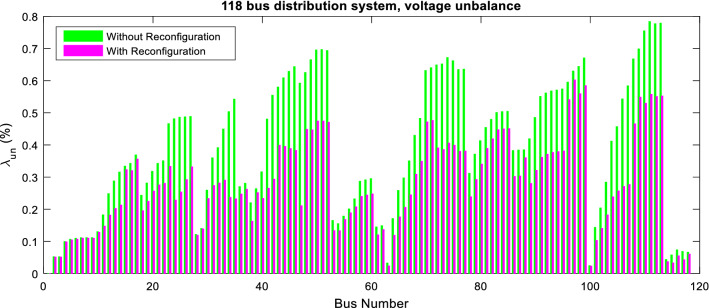
Voltage unbalance of 118-bus network via ITSOA with and without optimal configuration.

Figures 11 and 12 show variations of power quality indices using ITSOA without many and with-objective problems for the 118-bus network. As shown in Figs. 11 and 12, the voltage sag and unbalance values are decreased along with the network buses. So achieving optimal configuration based on multiobjective optimization. Moreover, the power loss and reliability of the power quality objectives are improved, too.

**Figure 13:**
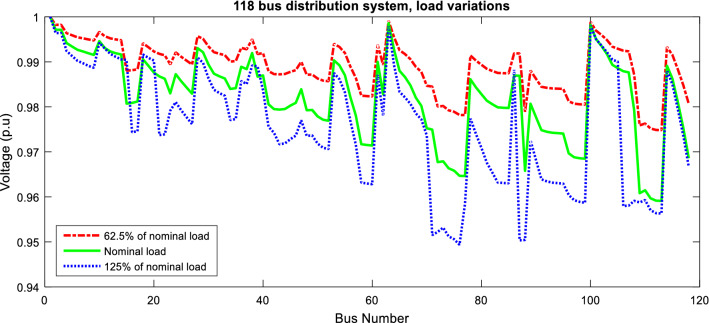
Voltage profile of 118-bus network with changing the demand via ITSOA with reconfiguration.

The effect of load variation of the 118-bus network is investigated in the optimization results. In Table 10, the results of these conditions are presented. The results, similar to the 33 and 69 bus networks, showed that the losses, voltage sag and unbalance and ENS are increased with increasing the demand, and vice versa. The power loss in light load (62.5% of nominal load), nominal load, and heavy load (125% of nominal load) is obtained at 401.773, 1050.481 and 1843.952 kW. The voltage profile of the 118-bus network is demonstrated by changing the demand. According to Fig. 13, the voltage profile of the network is enhanced by decreasing the demand and vice versa.

**Table 10:** Effect of changing the demand for the multiobjective problem of the unbalanced 118-bus network via ITSOA.

Method	Solution	Loss (kW)	$\lambda$_sag_ (p.u)	$\lambda$_un_ (%)	ENS (MWh/yr)
62.5% of nominal load	14, 23, 33, 39, 43, 47, 71, 85, 87, 95, 107, 108, 121, 123, 128	401.773	1.295	10.312	75.191
Initial	118, 119, 120, 121, 122, 123, 124, 125, 126, 127, 128, 129, 130, 131, 132	501.593	1.571	12.678	80.171
Nominal load	14, 23, 33, 39, 43, 47, 71, 85, 87, 95, 107, 108, 121, 123, 128	1050.481	2.0930	16.934	101.306
Initial	118, 119, 120, 121, 122, 123, 124, 125, 126, 127, 128, 129, 130, 131, 132	1322.47	2.549	23.098	128.274
125% of nominal load	15, 22, 33, 35, 39, 41, 71, 85, 86, 95, 107, 108, 121, 123, 128	1843.952	2.769	21.959	120.161
Initial	118, 119, 120, 121, 122, 123, 124, 125, 126, 127, 128, 129, 130, 131, 132	2109.637	3.218	26.882	150.343

### Comparison of the results with the previous research

The superiority of the ITSOA-based reconfiguration is compared with Ref.^46^ in Table 11, considering balanced and unbalanced networks. In^46^, the reconfiguration of the 118-bus balanced network is studied using PSO. In^47,48^, the optimal configuration of the 118-bus balanced system is developed with the objective of the power loss reduction using improved tabu search (ITS) and refined genetic algorithm (RGA), respectively. The results showed that the ITSOA has fewer losses than the PSO, ITS and RGA in a balanced network state, which proves the better capability of the ITSOA-based reconfiguration. Furthermore, the results showed that power loss was higher in unbalanced networks than in balanced networks.

**Table 11:** Results of balanced and unbalanced 118-bus network reconfigurations with active loss minimization.

Method	Opened switches	Loss (kW)
ITSOA (unbalanced)	14, 23, 33, 39, 43, 47, 71, 85, 87, 95, 107, 108, 121, 123, 128	882.27
ITSOA (balanced)	22, 23, 31, 33, 42, 52, 58, 71, 74, 95, 97, 109, 122, 129, 130	871.10
PSO (balanced)^46^	23, 26, 34, 39, 42, 51, 58, 71, 74, 95, 97, 109, 122, 129, 130	873.21
RGA (balanced)^47^	42,26,22,51,48,61, 39,127,73,72,76,82, 130,109,32	891.741
ITS (balanced)^48^	42,26,23,51,119,58, 39,95,74,71,97,129, 130,109,34	871.639

## Conclusion

In this paper, a new, improved transient search optimization algorithm (ITSOA) integrated with a multiobjective function is proposed to identify the optimal configuration of the unbalanced distribution network considering different loading. The optimal configuration of the network status of opened switches is determined using ITSOA and satisfying the constraints. The ITSOA is implemented to solve the multiobjective reconfiguration problem on unbalanced 13 and 118-bus networks. Simulation results, including power loss, power quality indices and energy not supplied, are evaluated before and after reconfiguration. The results showed that the ITSOA-based gaussian mutation achieved a better fuzzy fitness than the conventional TSOA. In addition, the results showed that multiobjective reconfiguration provides satisfactory results, compromising the different parts of the objective function. Furthermore, the results demonstrated that the best improvement was obtained for the voltage unbalance index, and the least improvement was observed for the reliability index. According to the load variation results, the increased demand weakened the power quality and reliability indices, and vice versa. The ITSOA was found to obtain better power quality and reliability indexes and with the lowest convergence tolerance with the highest speed and performance than conventional TSOA, PSO, GWO, BA, MRFO, and previous studies. Presenting a multi-objective optimization method based on a new improved algorithm and considering the compromise between multiple objectives to achieve the optimal configuration of distribution networks are the advantages of the proposed methodology compared to the existing network reconfiguration studies. The unbalanced network reconfiguration in conjunction with the allocation of renewable energy resources using a new hybrid optimization algorithm is suggested for future work.

## Data Availability

All data generated or analysed during this study are included in this published article.
